# Bone Marrow Niche Aging: Are Adipocytes Detrimental Cells in the Bone Marrow?

**DOI:** 10.3390/cells14110814

**Published:** 2025-05-30

**Authors:** Urban Švajger, Patrik Milić, Primož J. Rožman

**Affiliations:** 1 Slovenian Institute for Transfusion Medicine, Šlajmerjeva Cesta 6, 1000 Ljubljana, Slovenia; primoz.rozman@ztm.si; 2 Faculty of Pharmacy, University of Ljubljana, Aškerčeva Cesta 7, 1000 Ljubljana, Slovenia; patrik.milic@gmail.com

**Keywords:** bone marrow niche, aging, adipocytes

## Abstract

Aging disrupts the bone marrow (BM) niche, a complex microenvironment crucial for hematopoietic stem cell (HSC) maintenance. A key, yet debated, hallmark of this aging process is the accumulation of bone marrow adipocytes (BMAds). This review explores the evolving role of BMAds in the aging BM, particularly their influence on HSC regulation via metabolic, endocrine, and inflammatory pathways. Aging BMAds exhibit altered secretory profiles, including reduced leptin and adiponectin and increased pro-inflammatory signals, which skew hematopoiesis toward myeloid over lymphoid lineage production. Additionally, shifts in fatty acid composition and lactate signaling from BMAds may impair stem cell function. These changes, alongside aging-associated alterations in vascular, neural, and stromal components of the niche, contribute to diminished immune resilience in older adults. We discuss emerging therapeutic strategies targeting BMAd-derived factors, such as DPP4 inhibition or the modulation of β-adrenergic signaling, aimed at creating a more youthful BM environment. By summarizing current insights into the aging BM niche and the central role of BMAds, this review highlights mechanisms that could be targeted to rejuvenate hematopoiesis and improve immune function in the elderly.

## 1. Introduction

The mammalian immune system comprises lymphoid organs, namely the thymus, lymph nodes, and bone marrow (BM), along with various immune cell populations. The aging of the immune system, known as immunosenescence, contributes to increased morbidity in the elderly and is strongly associated with numerous age-related disorders. The progressive decline in immune function results in heightened susceptibility to infections, cancer, and autoimmune diseases, a diminished adaptive immune response following vaccination, and an elevated risk of degenerative conditions [1,2,3,4,5]. The term immunosenescence encompasses not only age-related alterations in immune cells, but also changes in the microenvironments or “niches” within lymphoid and non-lymphoid tissues that support immune cell function and maintain tissue homeostasis [6].

Immune cells originate from the hematopoietic system, which is hierarchically structured with hematopoietic stem cells (HSCs) at its apex. In adult humans, approximately 80 × 10^6^ HSCs reside in the BM [7], where they undergo self-renewal and differentiate into hematopoietic progenitors, which then further proliferate and mature into various blood cell lineages through the process of hematopoiesis [8,9]. Out of all HSCs, only 10% are steadily replicating [7] and produce 5 × 10^11^ hematopoietic cells daily, meaning each active HSC is participating in the daily production of 62,500 mature blood cells [10,11]. This capacity can expand in response to physiological demand [12]. Immune cells constitute approximately one-quarter of all mature blood cells [10], which are continuously replenished throughout an organism’s lifespan [13].

To sustain lifelong hematopoiesis, the self-renewal and differentiation of HSCs must be precisely regulated. Disruptions in these processes can result in HSC depletion or hematological disorders such as leukemia [14,15,16]. HSC fate is governed by intricate interactions between intrinsic and extrinsic regulatory factors [17,18]. Intrinsic factors include transcriptional regulators, epigenetic modifications, and metabolic pathways, which integrate extracellular cues. These extracellular signals are categorized into long-range signals (e.g., endocrine and neural signaling) and local signals derived from the BM microenvironment, collectively referred to as the “HSC niche”.

The concept of a “niche” as a regulatory unit for directing the HSCs’ fate decisions was first suggested by R. Schofield in 1978 [19]. Since then, the niche has been recognized as a complex and dynamic microenvironment in which interactions between HSCs, hematopoietic progenitor cells (HSPCs), and supporting niche cells modulate hematopoiesis. Niche cells influence HSC function through various signaling molecules, including cytokines and chemokines, which may be either secreted or membrane-bound. Additionally, extracellular matrix (ECM) components contribute to the formation of niches with distinct mechanical properties, affecting HSC regulation [20,21]. Moreover, recent evidence also suggests that metabolic products of the niche cells also have a significant impact on HSC regulation [22].

In this review, we will focus on age-related changes in the HSC microenvironment, with a particular emphasis on the role of bone marrow adipocytes (BMAds) and their influence on HSCs. BMAds accumulate progressively within the aging niche, potentially impacting hematopoietic function [23]. We will discuss how BMAds serve as active participants in the aging process, rather than passive fat deposits. By synthesizing current evidence on how aging-associated changes in BMAd characteristics impair HSC regulation, we will underscore their potential as novel therapeutic targets. This is of particular importance in the context of chemotherapy, transplantation, and age-related immune decline in the elderly.

For the sake of clarity, this paper will include nomenclature proposed by the Nomenclature Working Group of the International Bone Marrow Adiposity Society (BMAS) relevant to bone marrow adiposity (BMA) research [24].

## 2. The Bone Marrow Niche

Mammalian bone marrow (BM) cavities are highly vascularized and innervated, containing a complex network of non-hematopoietic and hematopoietic cells. The non-hematopoietic compartment consists primarily of bone marrow stromal cells (BMSCs) and their progeny, while the hematopoietic compartment includes hematopoietic stem cells (HSCs) and their descendants [25,26,27].

### 2.1. The Cellular Network of the Bone Marrow Niche

**Figure 1:**
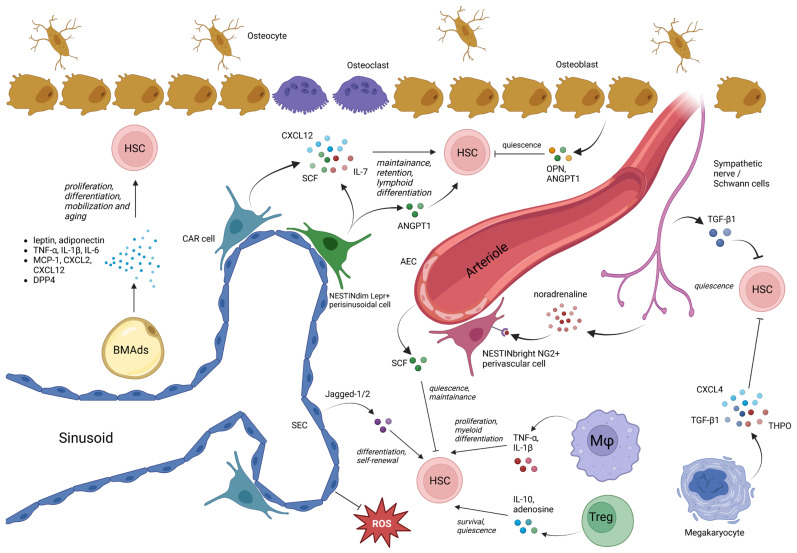
Cell types and their molecular interactions in the bone marrow (BM) niche. The BM niche comprises several cellular components, including vascular (arteriolar endothelial cells—AECs, sinusoidal endothelial cells—SECs), neural (sympathetic nerve fibers, Schwann cells), stromal (Nestinbright NG2+ perivascular cells, Nestindim LepR+ perisinusoidal cells, CXCL12-abundant reticular cells—CAR cells, osteoblasts, adipocytes—BMAds), and hematopoietic cells (hematopoietic stem cells—HSCs, megakaryocytes, macrophages—MΦ, regulatory T cells—Tregs). The figure shows critical niche-supportive signaling pathways, such as CXCL12, SCF, angiopoietin-1 (ANGPT1), osteopontin (OPN), thrombopoietin (THPO), transforming growth factor-beta 1 (TGF-β1), CXCL4, noradrenaline, and reactive oxygen species (ROS), regulating HSC maintenance, quiescence, differentiation, mobilization, and aging. BM adipocytes (BMAds) specifically modulate niche function through secreted adipokines and cytokines, significantly contributing to age-related BM niche alterations.

The regulation of HSCs involves a complex cellular network (Figure 1). Advances in genetically modified mouse models and microscopy techniques have identified various endogenous BM cell types capable of influencing HSC behavior through the secretion of niche-regulating molecules. These include CXC chemokine ligand 12 (CXCL12), stem cell factor (SCF), thrombopoietin (THPO), osteopontin (OPN), transforming growth factor-β (TGFβ), CXCL4, vascular cell adhesion molecule 1 (VCAM1), GP130, Notch ligands, fibroblast growth factor 1 (FGF1), and pleiotrophin (PTN). Cells that regulate HSCs through both molecular signaling and physical interactions are collectively referred to as “niche cells”. These cells can be categorized into four major components [28]:

1. The vascular component, comprising arteriolar endothelial cells (AECs) and sinusoidal endothelial cells (SECs).
2. The neural component, consisting of sympathetic nerves and non-myelinating Schwann cells.
3. The stromal component, including various perivascular BMSCs, osteoblasts, and adipocytes.
4. The hematopoietic component, encompassing HSCs and their progeny, such as phagocytic cells, megakaryocytes, and regulatory T cells (Tregs).

#### 2.1.1. The Vascular Component

A dense vascular network in BM ensures the delivery of growth factors, neurotransmitters, nutrients, hormones, and oxygen while facilitating the removal of metabolic waste. This vasculature comprises arteries supplying oxygenated blood, veins for blood exit, and an intermediary capillary network. In long bones like the femur, the nutrient artery enters the BM through the diaphysis and branches into central arteries, which further divide into thin-walled arterioles extending towards the metaphysis. These arterioles supply a capillary network composed of fenestrated, highly branched sinusoidal “type L” vessels. The transition from arterioles to sinusoids primarily occurs in the metaphysis and, to a lesser extent, in the diaphysis, mediated by “type H” vessels. Sinusoidal vessels converge into the central sinusoid, which spans the BM and drains into the nutrient vein, directing blood into systemic circulation [25,28].

Endothelial cells within the BM serve a critical role in HSC maintenance. In vivo studies demonstrate that the deletion of endothelial cell-specific genes, such as *Jag1* [29], *Cxcl12* [30,31], *Gp130* [32], or *Scf* [33] can disrupt HSC homeostasis. AECs and SECs, distinguished by their expression of SCA1 and podoplanin (PDPN), also show functional differences. AECs (CD45-Ter119-SCA1highPDPN−) produce nearly all endothelial cell-derived SCF, and its deletion significantly reduces functional HSCs, whereas SCF deletion in SECs (CD45-Ter119-SCA1dimPDPN+) does not yield comparable effects [34]. However, whether these results stem solely from SCF deletion in AECs or involve broader niche modifications remains unclear. Given the close association between endothelial cells and perivascular BMSCs, it is plausible that niche interdependencies contribute to these findings. Notably, SCF and CXCL12 levels in BM endothelial cells are considerably lower than in BMSCs [35,36].

Additionally, differences in vessel architecture impact HSC function. Although HSCs exhibit a hypoxic profile regardless of BM localization [37], recent studies indicate that vessel permeability influences local reactive oxygen species (ROS) levels, affecting HSC fate. AECs, which form less permeable vessels, associate with quiescent HSCs characterized by lower ROS levels, whereas SECs form more permeable vessels linked to higher ROS levels, promoting HSC activation and differentiation [38].

#### 2.1.2. The Neural Component

The skeleton is innervated by both branches of the autonomic nervous system: the sympathetic and parasympathetic nerve fibers [39,40,41]. Between the two, only sympathetic nerve fibers enter the BM [39,41]. It was shown that only 5% of all sympathetic nerve fibers reaching bone penetrate the BM [42]. Despite this, the BM has a higher density of autonomic nerves relative to the periosteum or mineralized bone [41]. Arterioles, located mainly near the endosteal regions of the BM, are cloaked by the sympathetic nerves that are covered by the non-myelinating Schwann cells. Both sympathetic nerves and non-myelinating Schwann cells have been labeled as niche cells due to their ability to regulate HSCs migration and quiescence either directly or indirectly through the production of the neurotransmitter noradrenaline and TGFβ [26].

Noradrenaline, released from sympathetic fibers, plays a crucial role in HSC mobilization by signaling through adrenergic receptors. Namely, the movement of HSCs from the BM into circulation depends on an intact gradient of CXCL12, which is disrupted by granulocyte colony-stimulating factor (G-CSF), leading to HSC mobilization [43,44]. The G-CSF-induced HSC mobilization was shown to be suppressed by the genetic or pharmacologic ablation of adrenergic neurotransmission, indicating that noradrenaline controls G-CSF-induced CXCL12 downregulation [45]. Interestingly, the G-CSF-induced migration of HSCs is linked to suppression of BMSC and osteolineage cell function [45,46,47,48,49]. Additionally, perivascular stromal cells, studied as Nes-GFP^+^ BMSCs, are directly targeted by sympathetic nerves. These cells respond to adrenergic signals via β3-adrenergic receptors, which regulate CXCL12 expression in a circadian manner and influence HSC egress in mice [46,50] and possibly in humans [51]. Human HSCs also express β2-adrenergic receptors, allowing for direct adrenergic regulation that promotes their migration and engraftment [52].

Non-myelinating Schwann cells, identified by glial fibrillary acidic protein (GFAP) expression, also contribute to HSC quiescence by activating the TGFβ/Smad signaling pathway in dormant HSCs. Surgical denervation via the transection of the postganglionic sympathetic nerve results in HSC depletion due to increased proliferation [53]. However, other denervation methods, such as acute surgical or chemical sympathectomy, do not significantly affect HSC numbers [46], which leaves questions open about how sympathetic signaling influences Schwann cells and whether other post-surgical effects, such as inflammation, could play a role.

Beyond sympathetic nerves, sensory fibers also penetrate the BM, though their role in hematopoiesis remains largely unknown [41,54,55,56,57]. Similarly, parasympathetic fibers have been observed in the distal femoral metaphysis [40,58], but their function in HSC regulation is unclear. However, evidence suggests that acetylcholine signaling through the type 1 muscarinic receptor (CHRM1) in the hypothalamus may regulate G-CSF-induced HSC mobilization. This process is mediated by the activation of the hypothalamic–pituitary–adrenal (HPA) axis, leading to glucocorticoid production, which in turn primes HSCs for migration [59].

#### 2.1.3. The Stromal Component

Bone marrow stromal cells (BMSCs) are a rare, self-renewing, and multipotent cell population that play a crucial role in maintaining the hematopoietic niche. These cells tightly surround arterioles while loosely associating with sinusoidal blood vessels. In vitro, they form colony-forming unit-fibroblasts (CFU-Fs) or non-adherent mesenspheres and can generate an organized hematopoietic niche with active hematopoiesis upon transplantation [60]. Their niche-organizing potential has been demonstrated through various transplantation experiments. For example, fetal mouse CD105^+^CD51^+^ BM stromal cells transplanted into the renal capsule of adult mice successfully induced ectopic hematopoietic marrow [61]. Similarly, human CD146^+^ BM stromal cells transplanted subcutaneously in mice formed heterotopic hematopoietic niches [62].

Sympathetic nervous system signaling plays a key role in HSC mobilization from the BM, primarily by regulating CXCL12 synthesis [45,46,51]. Within the stromal compartment, a subset of BMSCs, identified as perivascular cells by using green fluorescent protein (GFP) under the nestin promoter (Nes-GFP^+^ cells), was found to closely interact with nerves. These cells express high levels of niche factors essential for HSC maintenance and attraction, including CXCL12, SCF, ANGPT1, OPN, IL-7, and VCAM1 [50]. Similar populations have been identified in non-transgenic mice and human fetal BM using platelet-derived growth factor receptor-α (PDGFRα) and CD51 as markers [63]. Notably, Nestin^+^ BMSCs have been identified as essential constituents of the HSC niche, closely associated with HSCs and contributing to their maintenance and quiescence [50].

Another key BMSC subset, identified using a GFP knock-in at the *Cxcl12* locus, consists of CXCL12-abundant reticular (CAR) cells, which are mainly adipo-osteogenic progenitor cells located around sinusoids. CAR cells are primary producers of SCF and IL-7, critical for lymphoid progenitor and mature B cell maintenance [64,65,66]. Studies using a GFP knock-in at the *Scf* locus further identified perivascular cells marked by the adipo-osteogenic regulator LepR [33,67]. Around 90% of LepR^+^ BMSCs overlap with CAR BMSCs, and they also constitute about 80% of Nes-GFP^+^ cells [68,69,70].

Advanced 3D imaging and flow cytometry have identified two distinct subsets of Nes-GFP^+^ cells based on GFP expression and morphology. Nes-GFPbright cells, which are spindle-shaped NG2^+^ cells localized along arterioles, promote HSC quiescence, while Nes-GFPdim cells, with a reticular morphology, reside near sinusoids and overlap with LepR^+^ and CAR BMSCs [69]. The NG2+ BMSCs also express smooth-muscle marker MYH11 [36]. Depleting NG2^+^ BMSCs alters HSC localization, shifting them away from arterioles and leading to increased cycling, depletion from the BM, and reduced numbers in the spleen [69]. The selective deletion of *Cxcl12* in NG2^+^ periarteriolar cells—but not in sinusoidal LepR^+^ cells—also leads to HSC depletion and mislocalization [36]. Similarly, CAR BMSC depletion results in reduced HSC numbers, indicating their critical role in HSC retention [65]. The conditional deletion of *Cxcl12* in LepR^+^ BMSCs mobilizes HSCs to the circulation and spleen, but does not affect their numbers in the BM [33,36,71]. These findings suggest that distinct perivascular BMSC subsets regulate HSC maintenance through different cytokines (e.g., CAR or LepR+ cells versus NG2+ cells).

Earlier studies suggested that osteolineage cells play a role in HSC regulation [72,73,74], leading to the hypothesis that osteoblasts serve as a primary HSC niche [75,76,77,78]. However, more recent studies refute this, demonstrating that deleting *Cxcl12* or *Scf* in osteoblasts and osteoprogenitors has minimal impact on HSC numbers [30,31,33]. Although osteoblasts do not appear to be direct niche cells, they produce regulatory factors such as osteopontin (OPN), which negatively regulates HSC pool size [79,80], and angiopoietin 1 (ANGPT1) [81] and thrombopoietin (THPO) [82,83], which maintain HSC quiescence. However, hepatocytes, rather than osteoblasts, are now considered the primary source of functional THPO for HSC maintenance, while ANGPT1 is predominantly secreted by megakaryocytes, LepR^+^ BMSCs, and hematopoietic progenitors [35,84]. Additionally, studies show that quiescent HSCs are not preferentially located near osteoblasts [37,69]. Although osteolineage cells may not serve as primary HSC niche cells, they appear to support lymphoid progenitors [30,31,78,85,86]. In this context, osteoblast deficiency has been shown to severely impair hematopoiesis, underscoring the supportive role of osteoblasts in the HSC niche [87].

In contrast, adipolineage cells have emerged as functional niche cells. Bone marrow regions with high adipocyte content, such as mouse tail vertebrae, contain fewer HSCs compared to adipocyte-scarce regions like thoracic vertebrae. Studies in A-ZIP/F1 ‘fatless’ mice show improved HSC engraftment, suggesting that adipocytes negatively affect HSC function [88]. Blocking adipogenesis using peroxisome proliferator-activated receptor γ (PPARγ) antagonists enhances BM recovery after chemotherapy or transplantation [88,89]. Conversely, intratibial co-transplantation of adipogenic precursors with HSCs impairs engraftment and bone fracture healing, possibly due to excessive dipeptidyl peptidase-4 (DPP-4) production [90].

BM adipocytes also contribute to hematopoietic regeneration through SCF expression. In non-irradiated mice, SCF deletion from adipocytes reduces HSC frequency in tail vertebrae, which contain a high adipocyte density, but has little effect in long bones, where adipocytes are less abundant. Interestingly, A-ZIP/F1 ‘fatless’ mice show delayed hematopoietic recovery in long bones but not in tail vertebrae [91]. These findings suggest that BM adipocytes influence HSC maintenance and niche function, particularly in response to stress conditions such as irradiation or chemotherapy.

#### 2.1.4. The Hematopoietic Component

In addition to the vascular, neural, and stromal constituents of the hematopoietic stem cell (HSC) niche, HSC descendants themselves also play a role in regulating HSC function. Among the key hematopoietic regulators are megakaryocytes (Mk), phagocytic cells, and regulatory T (Treg) cells [28].

Megakaryocytes have been shown to directly regulate HSC quiescence [92,93,94,95]. Three-dimensional whole-mount imaging has revealed that HSCs are frequently localized near megakaryocytes, and selective depletion of Mk in vivo leads to HSC activation and proliferation [92]. A subpopulation of HSCs predisposed to megakaryocyte differentiation expresses platelet markers such as von Willebrand factor (vWF) and CD41 [96,97]. These vWF-GFP^+^ HSCs are preferentially positioned near Mk, and their depletion results in selective expansion, loss of lineage bias, and impaired long-term self-renewal following transplantation [98].

Megakaryocytes balance HSC quiescence through the secretion of the chemokine CXCL4 [92], TGF-β1 [93,99] and THPO [94,95]. In addition, CXCL4 and TGF-β1 secreted by Mk may restrict the formation of granulocyte/macrophage progenitor (GMP) clusters, an essential feature of emergency myelopoiesis, by enforcing HSC quiescence and thereby limiting the duration of the myelopoietic response [100].

Phagocytic cells, particularly BM macrophages, also influence HSC behavior. Macrophages regulate osteolineage cells and BMSCs to promote HSC retention while counteracting HSC egress induced by the autonomic nervous system [49,101,102]. In vivo depletion of macrophages resulted in HSC mobilization into the blood [102]. Moreover, in vivo and in vitro data suggest that BM CD169+ macrophages induce the expression of CXCL12 and other retention factors in Nes–GFP+ BMSCs to promote HSC retention [101], along with in vitro data showing that macrophages support osteoblast growth [49]. Meanwhile, BM monocytes contribute to HSC mobilization through granulocyte colony-stimulating factor (G-CSF)-receptor signaling [49].

Neutrophils influence HSC retention through their daily release and clearance in a circadian manner. Macrophages mediate this effect by clearing neutrophils via liver X receptor signaling [103]. Neutrophils also promote hematopoietic recovery after irradiation by producing TNF-α, which enhances vascular repair via TNF receptors [104]. This highlights both direct and indirect mechanisms by which phagocytic cells influence HSC behavior.

Recent findings suggest that BM dendritic cells (BMDCs) are an essential component of the perivascular hematopoietic niche. Most BMDCs exhibit a type 2 conventional dendritic cell (cDC2) phenotype, but RNA expression analysis suggests that they differ from splenic cDC2s, displaying unique expression patterns of chemokine receptors and toll-like receptors. BMDC depletion leads to the loss of BM macrophages and increased HSC mobilization, surpassing the effects of macrophage depletion alone. This suggests that BMDCs influence HSC mobilization through both macrophage-dependent and independent mechanisms. BMDC depletion also leads to endothelial expansion and increased vascular permeability, primarily due to CXCR2 upregulation in sinusoidal endothelial cells. In CXCR2-deficient mice, HSC mobilization following BMDC depletion is significantly reduced, confirming the role of BMDCs in sinusoidal permeability and HSC trafficking [105].

Regulatory T (Treg) cells also play a role in HSC regulation, as demonstrated in studies on allogeneic HSC (allo-HSC) transplantation. These studies suggest that Tregs promote HSC engraftment, survival, and quiescence [106,107,108,109]. Initial findings showed that certain T cell subsets improve HSC engraftment in allogeneic transplantation settings. Further studies revealed that allo-HSCs colocalize with FoxP3^+^ Tregs, which promote HSC survival by secreting the anti-inflammatory cytokine IL-10 [108]. Additionally, HSCs are closely associated with a subset of CD150high FoxP3^+^ Tregs, which express high levels of suppressive and costimulatory molecules compared to their CD150low counterparts. CD150high BM Tregs promote HSC quiescence by producing adenosine, catalyzed by CD39, a molecule highly expressed in this subset [109].

## 3. Aging of BM Niches

Aging leads to significant changes in the immune system, beginning with the decline of hematopoietic stem cells (HSCs) [110]. In mice, aging increases the number of phenotypically defined HSCs in the bone marrow (BM) but reduces their regenerative potential [111,112,113,114]. However, not all mouse strains exhibit an elevated quantity of HSCs. For instance, the amount of functional HSCs in BALB/c mice decreases with age [115]. Additionally, endogenous old HSCs are distributed away from their accepted niches [116], and have increased mobilization and diminished homing capacity into the BM [114,117,118,119,120]. When transplanted into young recipients, aged HSCs home further away from the endosteum compared to young HSCs [117]. Functionally, they exhibit a myeloid-biased differentiation pattern, producing fewer lymphoid cells while maintaining or even increasing myeloid lineage output [113,117,121]. While intrinsic, cell-autonomous mechanisms contribute to HSC aging [122], growing evidence suggests that changes in the BM niche also drive these age-related alterations [110].

### 3.1. Aging of the Hematopoietic Component

Aging affects the hematopoietic niche by altering its cellular composition. In aged mice, the BM contains increased numbers of megakaryocyte progenitors (MkP), megakaryocytes (Mk), platelets, and inflammatory M1-like macrophages (Mφ), which exhibit impaired phagocytic and efferocytic function—the ability to clear apoptotic cells [116,123]. The removal of apoptotic cells is an essential function of Mφs that avoids the necrosis of dead cells and prevents consequent local inflammation [124,125]. Moreover, engulfing apoptotic cells triggers the secretion of anti-inflammatory mediators in phagocytes [126]. This loss of efferocytic capacity leads to the accumulation of senescent neutrophils that produce IL-1β, a pro-inflammatory cytokine [123]. Chronic IL-1β exposure promotes the expansion of myeloid-biased CD41^+^ long-term HSCs (LT-HSCs) while enhancing myelopoiesis and suppressing lymphopoiesis [127].

### 3.2. Aging of the Vascular Component

Structural and functional changes occur in the BM vasculature with age. Although overall vascular density increases, the number of type-H capillaries and the length and number of arterioles decline [116,128]. Aged endothelial cells (ECs) exhibit higher permeability, increased reactive oxygen species (ROS) levels, greater hypoxia, and reduced expression of Jagged 1, CXCL12, and SCF. These changes impair niche function, as young HSCs co-cultured with aged ECs acquire a myeloid-biased differentiation profile and reduced engraftment potential. Conversely, aged HSCs co-cultured with young ECs partially regain function, demonstrating improved hematopoietic reconstitution, though they retain their myeloid bias [129]. These findings suggest that the aging of the vascular niche contributes to HSC dysfunction.

### 3.3. Aging of the Neural Component

Since arterioles and sympathetic nerves are closely linked, aging leads to a decline in arteriolar innervation. This is evidenced by a reduction in tyrosine hydroxylase-positive (TH^+^) adrenergic nerve fibers, β-III tubulin^+^ nerve fibers, and synaptic contacts between nerves and blood vessels [116]. The loss of sympathetic innervation disrupts circadian HSC egress, a feature preserved in young mice. Surgical BM denervation in young mice induces age-like microenvironmental changes, including shortened arteriolar vessels, increased CD31high ECs, and expanded CD51^+^ PDGFRα^+^ BMSCs with diminished clonogenic potential. These denervated BMSCs exhibit downregulated *Cxcl12*, *Scf*, and *Angpt1* expression, impaired osteogenic differentiation, and sustained adipogenic potential. The resulting HSC dysfunction includes impaired polarity, reduced engraftment, myeloid-biased differentiation, expanded HSC pools, and increased DNA damage (γH2AX foci) [116].

Sympathetic signaling via β2-adrenergic receptors (ADRβ2) and β3-adrenergic receptors (ADRβ3) plays a role in these aging-related changes [46,130,131]. While ADRβ3 expression remains stable in stromal cells with age, ADRβ2 is broadly expressed in both hematopoietic and non-hematopoietic compartments and declines over time [36,116,132]. The pharmacological activation of ADRβ2 or ADRβ3 in aged mice restores BMSC and EC numbers to youthful levels, but only ADRβ3 activation leads to functional HSC rejuvenation. ADRβ3 agonist treatment enhances long-term engraftment, multilineage reconstitution, and secondary transplantation success. Transcriptomic analysis confirms that ADRβ3 activation shifts aged HSC gene expression toward a youthful state. Additionally, it restores vascular and stromal structure, increasing the arteriolar density and clonogenic activity of CD51^+^ PDGFRα^+^ BMSCs while upregulating *Cxcl12* and *Scf* expression. Notably, young *Adrb3*−/− mice exhibit premature HSC aging, suggesting that neural dysfunction is a key driver of BM niche deterioration [116].

### 3.4. Aging of the Stromal Component

Aging alters perivascular BMSC populations, leading to an expansion of Nes-GFPbright perivascular BMSCs, which express NG2, a pericyte marker [69,116]. In contrast, the Nes-GFPdim sinusoidal BMSC population remains unchanged. Aged CD51^+^ PDGFRα^+^ stromal cells exhibit increased proliferation but reduced clonogenic potential, impaired osteogenesis, and increased adipogenic differentiation. These aged BMSCs also display lower expressions of key niche factors (CXCL12, SCF, and ANGPT1) [116]. Treatment with an ADRβ3 agonist partially restores stromal function, enhancing BMSC clonogenicity and niche factor expression, which in turn improves HSC function [116].

Osteolineage cells also undergo niche-related aging. Increased CCL5 expression in aged osteoblasts may contribute to HSC differentiation bias [133], while decreased osteopontin (OPN) expression is linked to impaired HSC function [134]. Treatment with thrombin-cleaved OPN reverses these defects, improving engraftment, restoring HSC polarity, and balancing lymphoid and myeloid differentiation [134].

## 4. Bone Marrow Adipocytes

**Figure 2:**
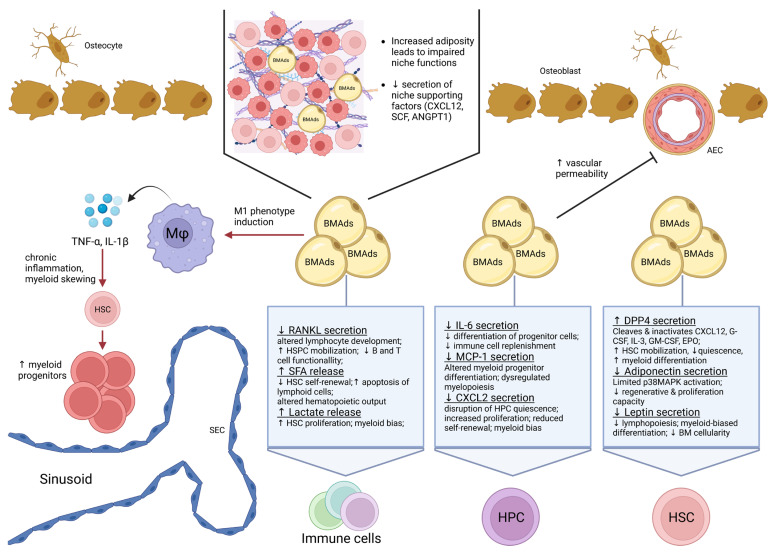
Impact of aged bone marrow adipocytes (BMAds) on the hematopoietic niche. Increased adiposity in the aging bone marrow negatively influences niche integrity and functionality, characterized by reduced secretion of essential niche-supporting factors (CXCL12, SCF, ANGPT1) from stromal cells and increased vascular permeability. Altered secretion profiles from aged BMAds directly affect hematopoietic stem cells (HSC), hematopoietic progenitor cells (HPC), and immune cell populations, causing disrupted hematopoiesis. Specific adipokines and secreted factors involved include decreased RANKL, IL-6, MCP-1, CXCL2, adiponectin, and leptin, alongside increased secretion of dipeptidyl peptidase-4 (DPP4), saturated fatty acids (SFAs), and potentially lactate. Additionally, aged BMAds promote a pro-inflammatory M1 macrophage phenotype, further exacerbating inflammation-driven myeloid skewing of HSC differentiation and reducing lymphopoiesis, thus significantly contributing to hematopoietic dysfunction associated with aging.

Regarding the effect of aging on adipocytes within the BM, this subject is still underexplored. Although the content of adipocytes in the BM increases with aging, their role in hematopoiesis remains a controversial topic [135,136] (Figure 2).

### 4.1. The Bone Marrow Adipose Tissue Ontogeny

In humans, bone marrow adipose tissue (BMAT) expands significantly after birth in a centripetal pattern. At birth, the BM consists primarily of hematopoietic cells and is referred to as red marrow due to its high erythrocyte content. As development progresses, adipocytes accumulate first in the distal skeleton (hands and feet), followed by the epiphyses of long bones, and later in the diaphysis—with distal regions accumulating fat more rapidly than proximal ones [137]. By the age of 25, 50–70% of BM volume is occupied by BMAT [138], contributing to the progressive shift from red to yellow marrow, which continues throughout the lifespan [139,140]. In the axial skeleton, adipocyte accumulation occurs later but is evident in the sternum, ribs, pelvis, and vertebrae by adulthood [141]. Within the spine, adiposity follows a gradient from the sacrum to the lumbar vertebrae, with spinal fat content increasing from 27% to 76% between the ages of 10 and 76 [142,143].

In rodents, BMAds also expand in a centripetal pattern. Early studies suggested that BM contains distinct adipocyte subtypes, differing in lipid composition and responses to metabolic changes [144]. This was later confirmed by Scheller et al. (2015), who classified BMAT into two distinct subtypes based on location and regulatory mechanisms: constitutive BMAT (cBMAT) and regulated BMAT (rBMAT) [145,146]. cBMAT develops early in life, localizing in distal tibia and caudal vertebrae. It consists of large, densely packed adipocytes enriched in unsaturated fatty acids and remains relatively stable despite metabolic or physiological changes. rBMAT forms later in proximal tibia, femur, and lumbar vertebrae, containing smaller, dispersed adipocytes with a higher content of saturated fatty acids (e.g., palmitate and stearate). This subtype is highly responsive to environmental factors [135].

A reduction in rBMAds was observed after prolonged cold exposure (4 °C) that lasted for three weeks [146], acute fasting [147], prolonged voluntary running [148], during lactation [149], and following intracerebral or subcutaneous leptin injection [150,151]. On the other hand, rBMAds boosters include a high-fat diet [148,152], caloric restriction (CR) [153,154,155], treatments with thiazolidinediones and glucocorticoids [156,157], fibroblast growth factor 21 (FGF21) [158], insulin-dependent diabetes mellitus (type I diabetes) [159], irradiation [91], and aging [90,160].

Both BMAT types are susceptible to extreme conditions, leading to gelatinous transformation of the BM (GTBM)—a rare hematologic condition associated with anorexia, systemic lupus erythematosus (SLE), malignancies, and HIV [161]. Additionally, hemorrhage leads to a decrease in both cBMAT and rBMAT [162].

### 4.2. Expression and Secretion of Bioactive Substances by BMAds

Adipocytes function as lipid storage cells and endocrine regulators, secreting adipokines, cytokines, chemokines, hormones, and metabolic byproducts (e.g., fatty acids and lactate), which influence inflammation and hematopoiesis [163,164,165]. While inflammation plays a role in tissue homeostasis, chronic inflammation disrupts tissue integrity, particularly with aging, where BMAT expansion contributes to immune dysregulation [166,167,168,169,170,171].

BMAds develop from BMSCs, and their differentiation hierarchy has been extensively reviewed elsewhere [136]. The in vitro differentiation of BMAds leads to an increased secretion of adipokines, including leptin, adiponectin, IL-6, MCP-1, RANKL, dipeptidyl peptidase-4 (DPP4), and resistin [90,172,173,174,175,176,177]. On the other hand, CXCL12 expression decreases significantly during adipocyte differentiation, while CXCL2 levels remain unchanged [178].

Aged BMAds exhibit altered cytokine and adipokine profiles. They show reduced leptin, adiponectin, and IL-6 expression, while TNF-α and IL-1β increase in middle-aged BMAds but decline in older BMAds [179]. Proteomic studies reveal age-related reductions in TNFα, IL-6, RANKL, CXCL2, and MCP-1, suggesting altered immune interactions [180].

### 4.3. Metabolic Products of BMAds and Their Impact on HSC Regulation

#### 4.3.1. Fatty Acids

BMAds regulate energy homeostasis through the storage and release of fatty acids. Triglycerides are stored under insulin regulation, while energy demand triggers lipolysis, leading to the breakdown of triglycerides into free fatty acids (FFAs) and glycerol [181,182]. In adipocytes, lipolysis is controlled by a complex system of lipases and lipid-binding proteins [183].

Among these, perilipins play a crucial role in adipocyte lipid metabolism by regulating access to triglyceride stores [184,185,186]. Perilipin 1 (PLIN1) modulates the activity of key lipases, such as adipose triglyceride lipase (ATGL) and hormone-sensitive lipase (HSL), under both basal and stimulated conditions [187]. Lipolysis can also occur at a basal level in the absence of external stimuli [188]. Elevated basal lipolysis has been observed in perilipin-null mice [189] and in patients with loss-of-function mutations in perilipin 1 [190]. Gene expression profiling of BMAds revealed that aged BMAds exhibit reduced PLIN1 expression, suggesting that increased basal lipolysis may lead to excessive exposure of the BM microenvironment to saturated fatty acids [179].

Fatty acid oxidation (FAO) has been proposed as a key metabolic process for HSC self-renewal and protection against exhaustion [191]. However, the role of different FA types in hematopoiesis appears to be more complex.

Higher monounsaturated fatty acid (MUFA) levels in BM extracellular fluid correlate with an increased proportion of long-term HSCs (LT-HSCs) and granulocyte-macrophage progenitors (GMPs), while common myeloid progenitors (CMPs) and megakaryocyte-erythrocyte progenitors (MEPs) remain unchanged. However, common lymphoid progenitors (CLPs) are reduced in this setting. Increased saturated fatty acid (SFA) content in BM extracellular fluid leads to a reduced number of LT-HSCs, while increasing the proportion of CLPs. Despite this early advantage for lymphopoiesis, peripheral B lymphocyte numbers remain unchanged. Additionally, higher SFA levels in BM extracellular fluid correlate with an increased rate of B and T lymphocyte apoptosis, leading to a net reduction in these immune cells in the BM [192].

These findings suggest that a higher MUFA-to-SFA ratio in BM extracellular fluid supports HSC maintenance and hematopoiesis, while elevated SFA levels negatively impact HSC self-renewal and immune cell survival.

#### 4.3.2. Lactate

Lactate is a key metabolic byproduct produced by adipocytes and other cell types. It acts as a signaling molecule by binding to G-protein-coupled receptor 81 (GPR81), which is also expressed on hematopoietic stem cells [165,193,194,195,196,197]. Despite its known systemic role, the contribution of bone marrow adipocytes (BMAds) to lactate production remains unclear. No direct studies have specifically examined lactate secretion from BMAds or the expression of glycolytic enzymes involved in glucose metabolism within these cells [198].

Unlike other adipose tissues, bone marrow adipose tissue (BMAT) does not respond to physiological insulin levels in terms of glucose uptake [199]. Additionally, recent findings suggest that adipocyte lactate production remains active even in insulin-resistant states, indicating a continuous metabolic output [164].

The role of lactate in hematopoiesis has been explored using *Gpr81*−/− mice, which lack lactate signaling. These mice showed a significant reduction in long-term HSCs (LT-HSCs), short-term HSCs (ST-HSCs), multipotent progenitors (MPPs), common myeloid progenitors (CMPs), and megakaryocyte-erythrocyte progenitors (MEPs) compared to wild-type controls. However, granulocyte-macrophage progenitors (GMPs) remained unchanged, and the knockout mice exhibited poor hematopoietic recovery following irradiation damage. Conversely, increasing serum lactate levels promoted myeloid progenitor expansion, suggesting that lactate plays a role in HSC proliferation and myeloid lineage differentiation [195]. These findings suggest that lactate plays a critical role in regulating HSC proliferation and differentiation, particularly favoring the myeloid lineage. Further research is required to fully understand how BMAds contribute to lactate metabolism and its impact on hematopoiesis.

### 4.4. The Impact of Adipokines Secreted Form BMAds on HSC Regulation and Hematopoiesis

Adipokines play a crucial role in hematopoiesis, with several secreted factors from bone marrow adipocytes (BMAds) influencing hematopoietic stem and progenitor cells (HSPCs). One key adipokine, adiponectin, which declines with age in BMAds, has been shown to stimulate HSPCs by binding to its receptors, activating the p38 MAPK pathway, and promoting proliferation while maintaining an immature state. This effect improves long-term hematopoietic reconstitution following transplantation into lethally irradiated mice [200]. Adiponectin-deficient mice exhibit reduced HSPC proliferation, prolonged bone marrow hypocellularity, and lower neutrophil and platelet counts following myelosuppressive injury, although steady-state hematopoiesis remains largely normal, apart from slightly elevated platelet levels [201,202].

Another critical adipokine, leptin, also declines with age and is involved in HSC regulation by binding to the leptin receptor [203]. In vitro, leptin stimulates myelopoiesis in both human and murine hematopoietic progenitor cells [204,205]. In vivo, leptin-deficient (ob/ob) mice exhibit increased circulating monocytes, reduced circulating lymphocytes, and impaired lymphopoiesis recovery following sub-lethal γ-irradiation [206,207]. Leptin receptor-deficient (db/db) mice show BM hypocellularity, impaired hematopoiesis, reduced lymphoid differentiation, and an age-related shift toward myeloid-biased differentiation [208,209].

Beyond these classical adipokines, BMAds secrete inflammatory cytokines, including IL-6, TNF-α, and IL-1β, which directly influence hematopoiesis. TNF-α and IL-1β, elevated in middle-aged BMAds but declining in aged BMAds, promote HSC proliferation and myeloid differentiation [210]. IL-6, which decreases with aging, is a crucial mediator of myeloid lineage development [211]. The chemokine C–C motif chemokine ligand 2 (CCL2/MCP-1), which declines with age, signals through C–C chemokine receptor type 2 (CCR2), affecting HSPCs and early myeloid progenitors [212]. While wild-type and CCR2-deficient BM cells exhibit similar abilities to form clonogenic myeloid progenitors in vitro, the addition of exogenous CCL2 does not enhance colony formation [213]. Some studies suggest that MCP-1 suppresses myeloid progenitor proliferation, as exogenous MCP-1 inhibits proliferation in murine and human BM myeloid progenitors [214,215]. In CCR2-deficient mice, myeloid progenitors exhibit an increased proliferative capacity, suggesting that MCP-1 may act as a negative regulator of myeloid expansion [216]. To date, no studies have specifically examined MCP-1 knockout models or MCP-1 blockade in HSC regulation.

Another chemokine, CXCL2 (macrophage inflammatory protein-2, MIP-2), which is secreted at lower levels in aged BMAds, binds to CXCR2, influencing HSC mobilization [217,218]. In vitro, CXCL2 suppresses the colony formation of myeloid progenitors in murine and human BM cells in a dose-dependent manner [215,219]. In vivo, *Cxcr2*−/− mice exhibit increased BM and spleen cellularity, along with elevated HSC and hematopoietic progenitor cell (HPC) numbers. These mice show impaired LT-HSC self-renewal, loss of quiescence, and reduced serial transplantation potential, while their myeloid differentiation is altered, leading to an expansion of granulocytes and granulocyte-macrophage progenitors (GMPs) [219,220]. Interestingly, lymphopoiesis is not significantly affected, as B and T cell numbers remain stable in the BM, spleen, and thymus [219]. CXCR2 also interacts with ligands other than CXCL2, making its precise role in HSC regulation complex [218,221].

RANKL, a TNF superfamily member, is another key regulator secreted at reduced levels by aged BMAds. It binds to RANK and competes with osteoprotegerin (OPG), which acts as its soluble decoy receptor, inhibiting RANKL signaling [222]. In RANKL-deficient mice, HSPC mobilization is increased, whereas OPG-deficient mice show reduced HSPC mobilization, indicating that RANKL plays a role in HSC retention [223]. Additionally, *RANK*−/− and *RANKL*−/− mice exhibit reduced splenic B lymphocytes, normal T cell populations, and active extramedullary hematopoiesis, highlighting its importance in lymphocyte development and function [224,225].

Another significant enzyme, dipeptidyl peptidase 4 (DPP4/CD26), expressed in BMAds, cleaves hematopoietic growth factors, altering HSC regulation [226]. Aged constitutive BMAds (cBMAds) express higher DPP4 levels, while regulated BMAds (rBMAds) show increased DPP4 secretion upon differentiation [90]. DPP4 enzymatic activity cleaves key hematopoietic regulators, including SDF-1 (CXCL12), G-CSF, IL-3, GM-CSF, and erythropoietin (EPO), impairing HSC retention and promoting myeloid-biased differentiation. Increased DPP4 activity correlates with HSC exhaustion, while the inhibition of DPP4 restores lymphoid differentiation, particularly in aged or stress-induced hematopoiesis models. The biological inhibitor of DPP4, tissue factor pathway inhibitor (TFPI), exerts its function through glypican-3 (GPC3), which is expressed on murine and human HSPCs. *Gpc3* knockout mice, which exhibit elevated DPP4 activity, display increased HSPC proliferation, reduced quiescence, and a smaller BM HSC pool, suggesting that DPP4-mediated cleavage promotes HSC exhaustion and mobilization [227]. Additionally, these knockout mice exhibit defects in monocytic/macrophage lineage development, aligning with the hematopoietic growth factors targeted by DPP4, which are essential for myeloid lineage differentiation [226,228].

These findings suggest that DPP4 activity in BMAds contributes to HSC aging by promoting stem cell mobilization, reducing quiescence, and shifting hematopoiesis toward a myeloid-dominant state, making DPP4 inhibition a potential therapeutic strategy to counteract age-related hematopoietic dysfunction.

Resistin, a small polypeptide hormone secreted by BMAds, has been largely overlooked in HSC regulation and hematopoiesis [229]. Recent studies suggest that resistin modulates BM progenitor cell proliferation, as recombinant mouse resistin enhances colony-forming unit (CFU) activity in a dose-dependent manner. In vivo, daily resistin injections increase total BM cellularity and promote cell cycle entry, as shown by a higher proportion of BM cells in S phase after five consecutive days of treatment. However, after ten consecutive days, this effect diminishes, and BM cellularity returns to baseline [122].

These findings indicate that resistin transiently drives BM progenitor cells out of quiescence into a proliferative state, but a homeostatic mechanism likely restores quiescence to prevent HSC exhaustion. The specific signaling pathways through which resistin affects HSCs remain poorly understood, and no further studies have explored its long-term impact on HSC maintenance, differentiation, or aging.

Altogether, the altered secretory profile of adipokines, cytokines, and chemokines in aged BMAds appears to contribute directly to HSC aging and their impaired ability to support immune function. These findings suggest that targeting age-related changes in BMAd signaling may provide new therapeutic avenues for restoring healthy hematopoiesis and immune system function in aging individuals.

While the overall number of adipocytes increases with age in the bone marrow, this quantitative expansion is accompanied by qualitative phenotypic changes that are equally critical to niche dysfunction [115]. Aging BMAds not only accumulate in number, but also exhibit hallmarks of cellular senescence, including the upregulation of senescence-associated β-galactosidase and a shift toward a pro-inflammatory secretory phenotype known as the senescence-associated secretory phenotype (SASP) [128]. This includes elevated levels of IL-6, TNF-α, and other cytokines that promote chronic inflammation, disrupt HSC quiescence, and bias hematopoiesis toward the myeloid lineage. Thus, it is not merely the increased adipocyte volume, but their senescence-driven secretory alterations that may actively impair hematopoietic regulation and accelerate immune aging.

### 4.5. A Potential Impact of BMAds on the HSC Niche Components

Aged BM carries an increased population of pro-inflammatory M1-like macrophages (Mφ), which show marked impairment in their phagocytic and efferocytic capacities [230]. Concurrently, vascular density in aged BM is elevated, but the vasculature becomes increasingly leaky and is composed of endothelial cells (ECs) with heightened reactive oxygen species (ROS) levels [231,232]. These vascular changes may, in part, be driven by M1-like Mφ, which produces elevated levels of IL-1β, a pro-inflammatory cytokine known to exert both pro-angiogenic effects and to increase vascular permeability [230,233].

In addition to their effect on immune cells, factors secreted by aged BM adipocytes (BMAds), including resistin, DPP4, FAs, and lactate, may further exacerbate vascular dysfunction. These molecules have been shown to promote vascular permeability [234,235,236,237,238]. Moreover, resistin and saturated FAs enhance ROS production [234,235,239,240]. Conversely, DPP4 inhibitors have been shown to exert vasculoprotective effects by reducing inflammation and oxidative stress, improving EC function, attenuating senescence and apoptosis, and increasing levels of circulating endothelial progenitor cells [241].

An open question still remains, namely, what drives the polarization of Mφ toward an M1-like phenotype with impaired efferocytosis in the aged BM? Evidence suggests that aged BMAds may play a role in this process. Classically activated M1 Mφ are characterized by low efferocytic capacity, while alternatively activated M2 Mφ are proficient in clearing apoptotic cells [242]. Several adipokines whose secretion declines in aged BMAds, including leptin, adiponectin, IL-6, MCP-1, and CXCL2, have been implicated in promoting M2 polarization and enhancing efferocytosis [243,244,245,246,247]. RANKL, also reduced in aged BMAds, has been shown to exert anti-inflammatory effects on macrophages in the presence of adipocyte-derived factors [248].

In contrast, increased DPP4 expression, as well as saturated FAs, have been shown to favor M1 macrophage polarization [247,249,250]. Although data on resistin remain limited, in vitro studies using human and murine monocytic/macrophage cell lines suggest that resistin may contribute to M1 polarization as well [249,251]. However, in vivo confirmation of these effects is currently lacking.

## 5. Conclusions

Aging profoundly affects the bone marrow niche. It alters its cellular and molecular composition in ways that compromise HSC function. One of the most significant changes is the expansion of BMAds, which not only replace the hematopoietic space but also actively contribute to inflammatory and metabolic changes in the niche microenvironment. HSC dysfunction is driven by a decline in supportive niche factors, increased production of inflammatory cytokines, and shifts in lipid metabolism.

Vascular changes exacerbate these effects further, as aged BM endothelial cells exhibit reduced expression of key niche factors, such as CXCL12 and SCF, while increasing vascular permeability and ROS levels. In a similar manner, the loss of sympathetic nerve innervation leads to impaired adrenergic signaling and the disruption of the circadian regulation of HSC migration, as well as the alteration of the balance of supportive stromal cells. These changes collectively create an environment that promotes HSC aging and deterioration.

The role of BM macrophages in HSC regulation is also altered with age, as their efferocytic capacity declines, which leads to local inflammation and increased IL-1β production. This inflammatory shift further skews hematopoiesis toward myeloid expansion, reinforcing the cycle of immune dysfunction. Meanwhile, adipocyte-derived factors such as DPP4, leptin, and adiponectin play critical roles in the modulation of HSC quiescence and differentiation. For this reason, their differential expression in aging BM contributes to hematopoietic imbalance.

Despite these detrimental changes, emerging therapeutic strategies offer potential avenues for intervention. The pharmacological modulation of β-adrenergic receptors has shown promise in restoring niche function and improving aged HSC engraftment. Targeting DPP4 or enhancing osteopontin signaling could mitigate age-associated hematopoietic defects, as well. While therapies like DPP4 inhibition and β-adrenergic modulation show promise in restoring aged BM function, they are not strictly BMAd-specific. Therefore, further research is needed to improve targeting specificity and assess long-term effects, as well as to identify novel therapeutic targets.

In conclusion, the aging BM niche undergoes profound structural and functional changes that impair HSC function and contribute to immune system decline. Understanding the intricate interactions between BMAds, vascular and neural components, and immunological factors will be essential for the future development of strategies to counteract age-related hematopoietic dysfunction and for improving immune resilience in older individuals.

## Data Availability

Not applicable.

## Abbreviations

The following abbreviations are used in this manuscript:

Abbreviation	Explanation
AECs	Arteriolar Endothelial Cells
ANGPT1	Angiopoietin-1
BM	Bone Marrow
BMAds	Bone Marrow Adipocytes
BMAS	Bone Marrow Adiposity Society
BMAT	Bone Marrow Adipose Tissue
BMA	Bone Marrow Adiposity
BMSCs	Bone Marrow Stromal Cells
CAR cells	CXCL12-abundant Reticular Cells
Cxcl12/CXCL12	C-X-C Motif Chemokine Ligand 12
cBMAT	Constitutive Bone Marrow Adipose Tissue
CD	Cluster of Differentiation
DPP4	Dipeptidyl Peptidase 4
ECM	Extracellular Matrix
ECs	Endothelial Cells
FA	Fatty Acid
FGF1	Fibroblast Growth Factor 1
GP130	Glycoprotein 130
HSCs	Hematopoietic Stem Cells
HSPCs	Hematopoietic Stem and Progenitor Cells
IL	Interleukin (e.g., IL-6, IL-1β)
LT-HSCs	Long-Term Hematopoietic Stem Cells
LepR	Leptin Receptor
Mφ/MΦ	Macrophage
NG2	Neural/Glial Antigen 2
OPN	Osteopontin
PDGFRα	Platelet-Derived Growth Factor Receptor Alpha
PTN	Pleiotrophin
RANKL	Receptor Activator of Nuclear Factor Kappa-Β Ligand
rBMAT	Regulated Bone Marrow Adipose Tissue
ROS	Reactive Oxygen Species
SCF	Stem Cell Factor
SECs	Sinusoidal Endothelial Cells
TGFβ/TGF-β1	Transforming Growth Factor Beta
THPO	Thrombopoietin
Tregs	Regulatory T Cells
VCAM1	Vascular Cell Adhesion Molecule 1
